# Non-contiguous finished genome sequence and contextual data of the filamentous soil bacterium *Ktedonobacter racemifer* type strain (SOSP1-21^T^)

**DOI:** 10.4056/sigs.2114901

**Published:** 2011-10-01

**Authors:** Yun-juan Chang, Miriam Land, Loren Hauser, Olga Chertkov, Tijana Glavina Del Rio, Matt Nolan, Alex Copeland, Hope Tice, Jan-Fang Cheng, Susan Lucas, Cliff Han, Lynne Goodwin, Sam Pitluck, Natalia Ivanova, Galina Ovchinikova, Amrita Pati, Amy Chen, Krishna Palaniappan, Konstantinos Mavromatis, Konstantinos Liolios, Thomas Brettin, Anne Fiebig, Manfred Rohde, Birte Abt, Markus Göker, John C. Detter, Tanja Woyke, James Bristow, Jonathan A. Eisen, Victor Markowitz, Philip Hugenholtz, Nikos C. Kyrpides, Hans-Peter Klenk, Alla Lapidus

**Affiliations:** 1 Oak Ridge National Laboratory, Oak Ridge, Tennessee, USA; 2 DOE Joint Genome Institute, Walnut Creek, California, USA; 3 Los Alamos National Laboratory, Bioscience Division, Los Alamos, New Mexico, USA; 4 Biological Data Management and Technology Center, Lawrence Berkeley National Laboratory, Berkeley, California, USA; 5 DSMZ - German Collection of Microorganisms and Cell Cultures GmbH, Braunschweig, Germany; 6 HZI – Helmholtz Centre for Infection Research, Braunschweig, Germany; 7 University of California Davis Genome Center, Davis, California, USA; 8 Australian Centre for Ecogenomics, School of Chemistry and Molecular Biosciences, The University of Queensland, Brisbane, Australia

**Keywords:** aerobic, heterotrophic, filamentous, non-motile, Gram-positive, moderately acidophilic, sporulating, transposon, broken-stick distribution, entropy, *Ktedonobacteraceae*, *Chloroflexi*, GEBA

## Abstract

*Ktedonobacter racemifer* corrig. Cavaletti *et al*. 2007 is the type species of the genus *Ktedonobacter*, which in turn is the type genus of the family *Ktedonobacteraceae*, the type family of the order *Ktedonobacterales* within the class *Ktedonobacteria* in the phylum ‘*Chloroflexi*’. Although *K. racemifer* shares some morphological features with the actinobacteria, it is of special interest because it was the first cultivated representative of a deep branching unclassified lineage of otherwise uncultivated environmental phylotypes tentatively located within the phylum ‘*Chloroflexi*’. The aerobic, filamentous, non-motile, spore-forming Gram-positive heterotroph was isolated from soil in Italy. The 13,661,586 bp long non-contiguous finished genome consists of ten contigs and is the first reported genome sequence from a member of the class *Ktedonobacteria*. With its 11,453 protein-coding and 87 RNA genes, it is the largest prokaryotic genome reported so far. It comprises a large number of over-represented COGs, particularly genes associated with transposons, causing the genetic redundancy within the genome being considerably larger than expected by chance. This work is a part of the *** G****enomic* *** E****ncyclopedia of* *** B****acteria and* *** A****rchaea * project.

## Introduction

Strain SOSP1-21^T^ (= DSM 44963 = NRRL B-41538) is the type strain of the species *Ktedonobacter racemifer*, which is the type species of the monotypic genus *Ktedonobacter*, the type genus of the family *Ktedonobacteraceae* [1]. *K. racemifer* was first described in 2006 [1,2] as an aerobic, non-motile, filamentous, mesophilic, Gram-positive heterotroph also capable of growing under microaerophilic conditions [1]. The genus name was derived from the Greek word *ktedon* -*onos*, fiber, and the Neo-Latin *bacter*, a rod, meaning a filamentous rod [1]. The species epithet is derived from the Latin adjective *racemifer*, carrying clusters of grapes [1]. The original spelling, *Ktedobacter racemifer was* corrected in 2007 on validation according to Rule 61 and Recommendation 6(7) [2]. Strain SOSP1-21^T^ was originally isolated from a soil sample of a black locust wood in Gerenzano, Northern Italy. Ten phylogenetically (class level) related strains were also isolated from soil samples collected at different locations in Northern Italy [1]. Only recently, a nearest cultivated neighbor, *Thermosporothrix hazakensis*, was isolated from hot compost in Japan [3]. Here we present a summary classification and a set of features for *K. racemifer* strain SOSP1-21^T^, together with the description of the complete genomic sequencing and annotation.

## Classification and features

Using NCBI BLAST [4], a representative genomic 16S rRNA sequence of *K. racemifer* SOSP1-21^T^ was compared under default settings (e.g., considering only the high-scoring segment pairs (HSPs) from the best 250 hits) with the most recent release of the Greengenes database [5] and the relative frequencies of taxa and keywords (reduced to their stem [6]) were determined, weighted by BLAST scores. The most frequently occurring genus was *'Ktedobacter'* (100.0%) (1 hit in total; this represents the original, incorrect spelling of *Ktedonobacter*). No hits to sequences with (other) species names were found. (Note that the Greengenes database uses the INSDC (= EMBL/NCBI/DDBJ) annotation, which is not an authoritative source for nomenclature or classification.) The highest-scoring environmental sequence was AM180157 ('New lineage filamentous spore-forming soil isolate SOSP1-30SOSP1-30 str. SOSP1-30'), which showed an identity of 99.0% and an HSP coverage of 95.2%. The most frequently occurring keywords within the labels of environmental samples which yielded hits were 'soil' (11.2%), 'prari, tallgrass' (4.9%), 'miner, weather' (1.9%), 'new' (1.8%) and 'filament, lineag, spore-form' (1.6%) (249 hits in total). These keywords reflect some of the ecological properties reported for strain SOSP1-21^T^ in the original description [1]. Environmental samples which yielded hits of a higher score than the highest scoring species were not found.

**Figure 1:**
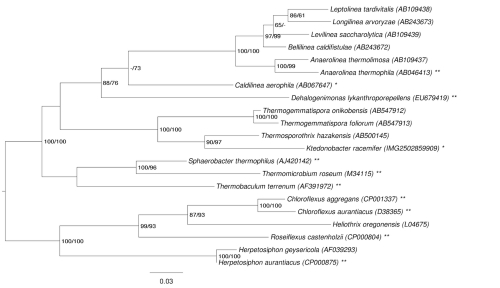
Phylogenetic tree highlighting the position of *K. racemifer* relative to the other type strains within the phylum ‘*Chloroflexi*’. The tree was inferred from 1,359 aligned characters [7,8] of the 16S rRNA gene sequence under the maximum likelihood (ML) criterion [9]. Rooting was done initially using the midpoint method [10] and then checked for its agreement with the current classification (Table 1). The branches are scaled in terms of the expected number of substitutions per site. Numbers above branches are support values from 750 ML bootstrap replicates [11] (left) and from 1,000 maximum parsimony bootstrap replicates [12] (right) if larger than 60%. Lineages with type strain genome sequencing projects registered in GOLD [13] are labeled with one asterisk, those also listed as 'Complete and Published' with two asterisks [14-17] as well as CP001337, CP000804, CP000909, CP002084, and AP012029.

Figure 1 shows the phylogenetic neighborhood of *K. racemifer* in a 16S rRNA based tree. The sequences of the eight 16S rRNA genes copies in the genome differ by up to nine nucleotides from each other and by up to five nucleotides from the previously published 16S rRNA sequence (AM180156), which contains two ambiguous base calls.

**Table 1:** Classification and general features of *K*. *racemifer* SOSP1-21^T^ according to the MIGS recommendations [18] and the NamesforLife database [19]

MIGS ID	Property	Term	Evidence code
	Current classification	Domain *Bacteria*	TAS [20]
		Phylum *Chloroflexi*	TAS [21,22]
		Class *Ktedonobacteria*	TAS [1-3]
		Order *Ktedonobacterales*	TAS [1,2]
		Family *Ktedonobacteraceae*	TAS [1,2]
		Genus *Ktedonobacter*	TAS [1,2]
		Species *Ktedonobacter racemifer*	TAS [1]
		Type strain SOSP1-21	TAS [1]
	Gram stain	positive	TAS [1]
	Cell shape	filamentous	TAS [1]
	Motility	non-motile	TAS [1]
	Sporulation	spherical spore-forming	TAS [1]
	Temperature range	mesophile	TAS [1]
	Optimum temperature	28-33°C	TAS [1]
	Salinity	NaCl up to 10 g/l growth w/o problem, inhibited at 30 g/l	TAS [1]
MIGS-22	Oxygen requirement	aerobic and microaerophilic	TAS [1]
	Carbon source	sugars and peptides	TAS [1]
	Energy metabolism	heterotrophic	TAS [1]
MIGS-6	Habitat	soil	TAS [1]
MIGS-15	Biotic relationship	free-living	NAS
MIGS-14	Pathogenicity	none	NAS
	Biosafety level	1	TAS [23]
	Isolation	soil from a black locust wood	TAS [1]
MIGS-4	Geographic location	Gerenzano, Northern Italy	TAS [1]
MIGS-5	Sample collection time	November 2001	NAS
MIGS-4.1	Latitude	45.64	NAS
MIGS-4.2	Longitude	9.00	NAS
MIGS-4.3	Depth	not reported	
MIGS-4.4	Altitude	about 210 m	NAS

Evidence codes - TAS: Traceable Author Statement (i.e., a direct report exists in the literature); NAS: Non-traceable Author Statement (i.e., not directly observed for the living, isolated sample, but based on a generally accepted property for the species, or anecdotal evidence). These evidence codes are from of the Gene Ontology project [24].

*K. racemifer* strain SOSP1-21^T^ cells are rod-shaped, filamentous and grow both vegetative and aerial mycelia on solid medium (Figure 2a). The large aerial hyphae produce spherical spores that cluster together with a grape-like appearance (Figure 2b). All other *K. racemifer* strains produced rounded spores, although they were arranged differently on the aerial hyphae [1]. Filamentous growth of strain SOSP1-21^T^ also occurred in submerged cultures, which contained the branched mycelia known from actinomycetes [1]. SOSP1-21^T^ stains Gram-positive and is not acid fast [1]. It produces pigments ranging from cream to pinkish orange on all media [1]. Although essentially aerobic, SOSP1-21^T^ is capable of growing under microaerophilic conditions [1]. The optimal growth temperature is 28-33°C [1]. It grows well at pH values between 4.8 and 6.8 with an optimum at pH 6 [1]. Salinity up to 10 g per liter does not inhibit the growth of the strain [1].

**Figure 2a:**
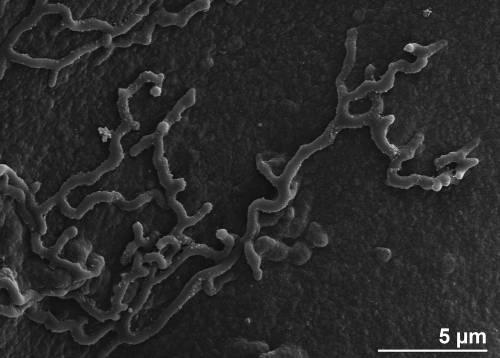
Scanning electron micrographs of *K*. *racemifer* SOSP1-21^T^ mycelium.

**Figure 2b:**
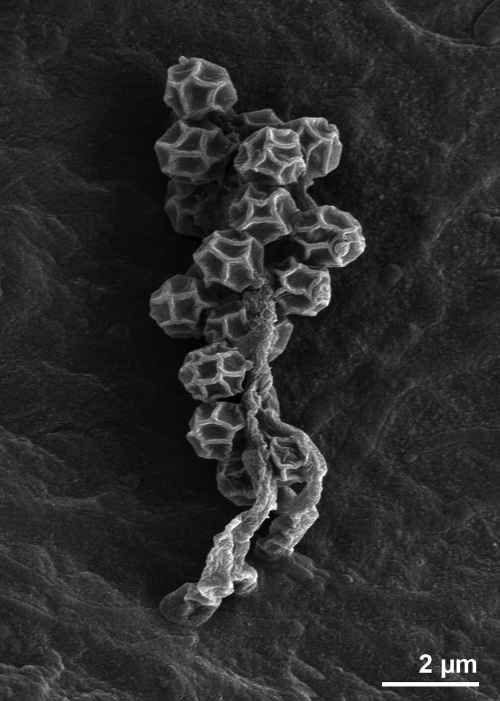
Scanning electron micrographs of *K*. *racemifer* SOSP1-21^T^ spores.

Strain SOSP1-21^T^ was capable of hydrolyzing starch, casein, gelatin, and (to a lesser extent) keratin but not cellulose, xylan, or chitin [1]. Strain SOSP1-21^T^ was catalase positive and produced H_2_S but could not reduce nitrates [1]. It is sensitive to 5 ug/ml novobiocin or ramoplanin and to 20 mg/ml apramycin and the glycopeptide A40926.

### Chemotaxonomy

The peptidoglycan of strain SOSP1-21^T^ contains ornithine, alanine, glutamic acid, serine, and glycine at a molar ratio of approximately 0.7:1.8:1.0:0.8:1.9 [1]. Serine was identified at the N-terminus of the interpeptide bridge [1]. When originally described, a detailed peptidoglycan structure had not been determined but A-type cross-linkage was suggested [1]. The cellular fatty acid pattern of strain SOSP1-21^T^ was reported to be characterized by an unusual high abundance of C_16:1 2-OH_ (30%) with other dominant lipids being branched-chain saturated fatty acids *iso*-C_17:0_ (25%), *iso*-C_16:0_ (11.5%) and *anteiso*-C_17:0_ (9.6%), as well as C_16:0 10-Me_ (7.8%) and C_16:0_ (6.7%) [1]. Our own data (DSMZ) did not confirm this fatty acid spectrum, but revealed *iso*-C_16:0_ (20.1%) as the most frequent fatty acid, followed by *anteiso*-C_17:0_ (18.5%), *iso*-C_17:0_ (15.0%), only 13.1% C_16:1 2-OH_ and 11.6% C_16:0 10-Me_. Polar lipids consisted of phosphatidylinisitol, phosphatitylglycerol, diphosphatidylglycerol and an unknown glycolipid [1]. MK-9(H__2__) was the only menaquinone reported for strain SOSP1-21^T^ [1].

## Genome sequencing and annotation

### Genome project history

This organism was selected for sequencing on the basis of its phylogenetic position [25], and is part of the *** G****enomic* *** E****ncyclopedia of* *** B****acteria and* *** A****rchaea * project [26]. The genome project is deposited in the Genomes OnLine Database [13] and the complete genome sequence is deposited in GenBank. Sequencing, finishing and annotation were performed by the DOE Joint Genome Institute (JGI). A summary of the project information is shown in Table 2.

**Table 2:** Genome sequencing project information

**MIGS ID**	**Property**	**Term**
MIGS-31	Finishing quality	Non-contiguous finished
MIGS-28	Libraries used	Two Sanger 8 kb pMCL200 and fosmid libraries; one 454 pyrosequence standard library
MIGS-29	Sequencing platforms	ABI3730, 454 GS FLX
MIGS-31.2	Sequencing coverage	10.1 × Sanger; 24.6 × pyrosequence
MIGS-30	Assemblers	Newbler version 1.1.02.15, phrap
MIGS-32	Gene calling method	Prodigal 1.4, Genemark 4.6b, tRNAScan-SE-1.23, infernal 0.81
	INSDC ID	ADVG00000000
	Genbank Date of Release	June 14, 2010
	GOLD ID	Gi02261
	NCBI project ID	27943
	Database: IMG-GEBA	648276680
MIGS-13	Source material identifier	DSM 44963
	Project relevance	Tree of Life, GEBA

### Growth conditions and DNA isolation

*K. racemifer* SOSP1-21^T^, DSM 44963, was grown in DSMZ medium 65 (GYM *Streptomyces* medium) [27] adjusted to pH 6.0, at 28°C. DNA was isolated from 0.5-1 g of cell paste using Qiagen Genomic 500 DNA Kit (Qiagen 10262) following the manufacturer’s protocol, with cell lysis protocol st/LALMP as described in Wu *et al*. [26]. DNA is available through the DNA Bank Network [28].

### Genome sequencing and assembly

The genome was sequenced using a combination of Sanger and 454 sequencing platforms. All general aspects of library construction and sequencing can be found at the JGI website [29]. Pyrosequencing reads were assembled using the Newbler assembler (Roche). The initial Newbler contigs were broken into 14,080 overlapping fragments of 1,000 bp and entered as pseudo-reads into the subsequence assembly. The sequences were assigned quality scores based on Newbler consensus q-scores with modifications to account for overlap redundancy and to adjust inflated q-scores. A hybrid 454/Sanger assembly was produced using parallel phrap (High Performance Software, LLC). Possible mis-assemblies were corrected with Dupfinisher [30], or transposon bombing of bridging clones (Epicentre Biotechnologies, Madison, WI) [31]. Some gaps between contigs were closed by editing in Consed [32], custom primer walking or PCR amplification. A total of 3,354 Sanger finishing reads and five shatter libraries were produced to close gaps, to resolve some repetitive regions, and to raise the quality of the finished sequence. Illumina reads were also used to correct potential base errors and increase consensus quality using a software Polisher developed at JGI [33]. The error rate of the completed genome sequence is less than 1 in 100,000. Together, the combination of the Sanger and 454 sequencing platforms provided 34.7 × coverage of the genome. The final assembly contained 165,050 pyrosequence and 2,305,667 Illumina reads.

### Genome annotation

Genes were identified using Prodigal [34] as part of the Oak Ridge National Laboratory genome annotation pipeline, followed by a round of manual curation using the JGI GenePRIMP pipeline [35]. The predicted CDSs were translated and used to search the National Center for Biotechnology Information (NCBI) non-redundant database, UniProt, TIGR-Fam, Pfam, PRIAM, KEGG, COG, and InterPro databases. Additional gene prediction analysis and functional annotation were performed within the Integrated Microbial Genomes - Expert Review (IMG-ER) platform [36].

## Genome properties

**Figure 3:**
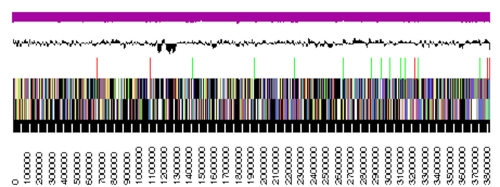
Graphical linear map of the largest, 3,837,106 bp long contig. From bottom to the top: Genes on forward strand (color by COG categories), Genes on reverse strand (color by COG categories), RNA genes (tRNAs green, rRNAs red, other RNAs black), GC content, GC skew.

The non-contiguous finished genome consists of ten contigs ranging in size from 1,579 bp to almost four Mbp, with five contigs being longer than one Mb (1,302,518 bp, 2,713,222 bp, 2,766,182 bp, 2,916,502 bp, and 3,837,106 bp) and a G+C content of 53.8% (Table 3 and Figure 3). Of the 11,540 genes predicted, 11,453 were protein-coding genes, and 87 RNAs; No pseudogenes were identified. The majority of the protein-coding genes (61.2%) were assigned a putative function while the remaining ones were annotated as hypothetical proteins. The distribution of genes into COGs functional categories is presented in Table 4.

**Table 3:** Genome Statistics

**Attribute**	**Value**	**% of Total**
Genome size (bp)	13,661,586	100.00%
DNA coding region (bp)	10,422,932	76.29%
DNA G+C content (bp)	7,348,426	53.79%
Number of contigs	10	
Extrachromosomal elements	unknown	
Total genes	11,540	100.00%
RNA genes	87	0.75%
rRNA operons	8	
Protein-coding genes	11,453	99.25%
Pseudo genes	0	
Genes with function prediction	7,065	61.22%
Genes in paralog clusters	4,919	42.63%
Genes assigned to COGs	6,654	57.66%
Genes assigned Pfam domains	7,250	62.82%
Genes with signal peptides	2,660	23.05%
Genes with transmembrane helices	2,581	22.27%
CRISPR repeats	7	

**Table 4:** Number of genes associated with the general COG functional categories

Code	value	%age	Description
J	224	2.9	Translation, ribosomal structure and biogenesis
A	0	0.0	RNA processing and modification
K	893	11.6	Transcription
L	975	12.6	Replication, recombination and repair
B	3	0.0	Chromatin structure and dynamics
D	34	0.4	Cell cycle control, cell division, chromosome partitioning
Y	0	0.0	Nuclear structure
V	215	2.8	Defense mechanisms
T	617	8.0	Signal transduction mechanisms
M	257	3.3	Cell wall/membrane/envelope biogenesis
N	20	0.3	Cell motility
Z	0	0.0	Cytoskeleton
W	0	0.0	Extracellular structures
U	54	0.7	Intracellular trafficking, secretion, and vesicular transport
O	195	2.5	Posttranslational modification, protein turnover, chaperones
C	416	5.4	Energy production and conversion
G	612	7.9	Carbohydrate transport and metabolism
E	474	6.2	Amino acid transport and metabolism
tF	135	1.8	Nucleotide transport and metabolism
H	264	3.4	Coenzyme transport and metabolism
I	236	3.1	Lipid transport and metabolism
P	255	3.3	Inorganic ion transport and metabolism
Q	217	2.8	Secondary metabolites biosynthesis, transport and catabolism
R	1,098	14.4	General function prediction only
S	519	6.7	Function unknown
-	4,886	42.3	Not in COGs

## Insights from the genome sequence

### Genome structure

**Figure 4:**
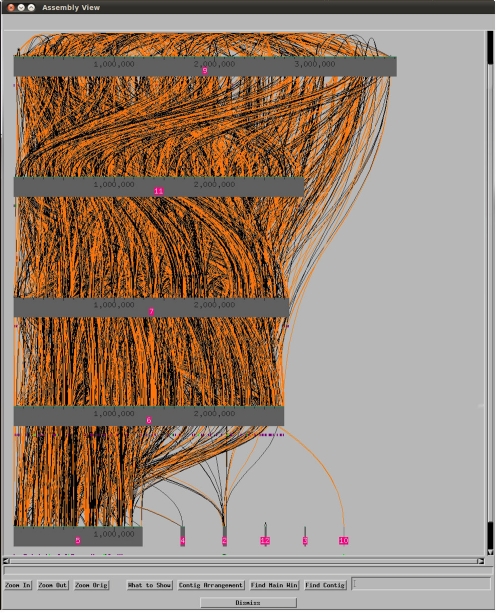
Screen shot from CROSSMATCH [32] indicating the matches between sequences within and across the contigs. CROSSMATCH options were – minmatch 30 – minscore 60.

With a length of 13,661,586 bp for the ten contigs (Table 3) *K. racemifer* SOSP1-21^T^ has the largest of all completely sequenced 1,760 archaeal and bacterial genomes [37] thus far, followed by *Sorangium cellulosum*, 13.0 Mbp [38], *Steptomyces bingchenggensis*, 11.9 Mbp [39], *Catenulispora acidiphila*, 10.5 Mbp [40], and *Streptosporangium roseum*, 10.4 Mbp [41]. However, this genome was also one of the most difficult to assemble. Figure 4 shows the unusually high number of identical sequence fragments across the genome, which caused the termination of the project as non-contiguous finished genome without closure of the last ten sequence gaps.

## Comparative genomics

Lacking an available genome sequence of the closest relative of *K. racemifer, Thermosporothrix hazakensis* [3] (Figure 1), the following comparative analyses were done with *Sphaerobacter thermophilus* [42] and *Thermomicrobium roseum* [43], the closest organisms phylogenetically for which there are publically available genome sequences [15,16].

*K. racemifer* stands out because of its enormous genome size of more than 13 Mbp. The genomes of *S. thermophilus* and *T. roseum* are significantly smaller, 3.9 Mbp and 2.9 Mbp, respectively. Whereas *S. thermophilus* and *T. roseum* have similar G+C-contents of 68% and 64%, respectively, the G+C-content of the *K. racemifer* genome is significantly lower (54%).

**Figure 5:**
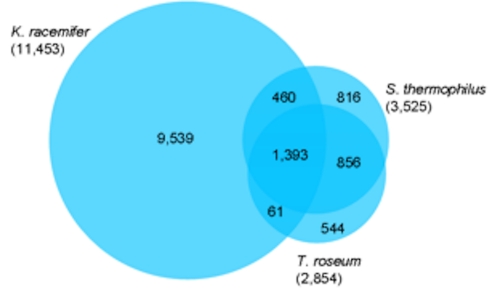
Venn diagram depicting the intersections of protein sets (total number of derived protein sequences in parentheses) of *K. racemifer*, *S. thermophilus* and *T. roseum.*

The fraction of shared genes in the three genomes is shown in a Venn diagram (Figure 5). The numbers of pairwise shared genes were calculated with the phylogenetic profiler function of the IMG-ER platform [36]. Homologous genes within the genomes were detected with a maximum E-value of 10^-5^ and a minimum identity of 30%.

A total of 1,393 genes are shared by the three genomes, referring to the whole genome sizes 39% and 48% of the genes in *S. thermophilus* and *T. roseum* have homologs in the three genomes, in the case of *K. racemifer* only 12% of the genes are shared by the other two genomes. The pairwise comparison of *S. thermophilus* and *T. roseum* revealed 2,249 genes which are shared by these two organisms, referring to the whole genomes 64% of the *S. thermophilus* genes and 79% of the *T. roseum* have homologous genes in the respective other genome.

The genome of *K. racemifer* encodes an enormously high number of transposon-associated genes; its annotation revealed 601 genes encoding transposases, 151 genes encoding integrases and 107 genes encoding resolvases. The genes coding these enzymes are spread over the whole genome with some regions having a higher density than others. The extremely high number of transposases is due to several gene copies that are to a greater or lesser extent similar in their sequences. The presence of that many mobile elements may explain the unusually high number of identical sequence fragments across the genome and the resulting difficulties occurring during the genome assembly.

Within the 9,539 unique genes of *K. racemifer* that have no detectable homologs in the genomes of *S. thermophilus* and *T. roseum* (under the sequence similarity thresholds used for the comparison) the 29 genes encoding xylose isomerases appear to be especially noteworthy; for 27 of these isomerase genes no homologous genes were detected in the other two genomes; only one gene was identified in *T. roseum*, and two in *S. thermophilus*. The high number of xylose isomerase genes suggests a strong utilization of pentoses by *K. racemifer*. To date *K. racemifer* was not tested regarding xylose utilization, but the close relative *T. hazakensis* is able to use xylose as the only carbon source [3]. Furthermore, a high number of genes encoding proteins responsible for resistance against several antibiotics were predicted: 61 bleomycin resistance proteins and 41 aminoglycoside phosphotransferases.

An estimate of the overall similarity between *K. racemifer*, *S. thermophilus* and *T. roseum*, was generated with the GGDC Genome-to-Genome Distance Calculator [44,45]. This system calculates the distances by comparing the genomes to obtain HSPs (high-scoring segment pairs) and interfering distances from a set of formulas (1, HSP length / total length; 2, identities / HSP length; 3, identities / total length). Table 5 shows the results of the pairwise comparison between the three genomes.

**Table 5:** Pairwise comparison of *K. racemifer*, *S. thermophilus* and *T. roseum* using the GGDC-Calculator.

		HSP length /total length [%]	identities /HSP length [%]	identities /total length [%]
*K. racemifer*	*S. thermophilus*	0.57	86.4	0.50
*K. racemifer*	*T. roseum*	0.48	87.2	0.42
*T. roseum*	*S. thermophilus*	9.41	83.1	7.82

The pairwise comparison (Table 5) of the genomes of *K. racemifer* with *S. thermophilus* and *T. roseum* revealed that only 0.57% and 0.48% of the average of the genome lengths are covered with HSPs. The identity within these HSPs was 86.4% and 87.2%, whereas the identity over the whole genome was only 0.50% and 0.42%, respectively. The comparison of *T. roseum* with *S. thermophilus* revealed that 9.41% of the average of both genome lengths are covered with HSPs, with an identity within these HSPs of 83.1%. The identity over the whole genome is 7.82%. These results show how distant the relationship between *K. racemifer* and *S. thermophilus* and *T. roseum,* respectively, is, if genome sizes are taken into consideration.

In order to quantify the differences in gene redundancy between the three genomes, as well as to determine over-represented genes, we used approaches based on entropy and the broken-stick distribution, respectively, applied to the set of genes from either genome assigned to COGs. Shannon's entropy (see, e.g., pp. 214, 243 in [46]) *H* can be used as a measure of disorder for discrete distributions; it is maximum (*H_max_*) if all categories (COGs in our case) are represented by exactly one item (gene) and then equal to the logarithm of the number of items (genes). Thus, one can measure the evenness (non-redundancy) within such a distribution as *H/H_max_* and the corresponding redundancy as *1.0 – H/H_max_*. The broken-stick distribution reflects the relative abundance of a given number of categories within a random population of items (see, e.g., p. 244 and 410 in [46]). Over-represented items (here: COGs) are those whose real relative frequencies (here: number of genes assigned to this COG relative to the total number of genes assigned to COGs) are larger than the broken-stick value of the corresponding rank within the list of frequencies sorted in decreasing order. Moreover, the entropy *H_exp_* of the broken-stick distribution can be used as an estimate for the expected entropy, yielding *1.0 – H/H_exp_* as an alternative measure of redundancy (which becomes negative when the evenness is larger than expected by chance).

The 2,022 genes assigned to 1,300 distinct COGs in the genome of *T. roseum* corresponded to an entropy of 6.912, an expected entropy of 6.748 and, hence, a redundancy of 9.20% if measured using *H_max_* and of -2.42% using *H_exp_*, whereas *S. thermophilus* (2,619 genes assigned to 1,383 COGs) yielded an entropy of 6.837 (expected: 6.810) and a redundancy of 13.14% with *H_max_* and -0.39% with *H_exp_*. In contrast, the 6,654 genes assigned to 1,731 distinct COGs in the genome of *K. racemifer* yielded an entropy of only 6.455 (expected: 7.034) and a redundancy of 26.67% (using *H_max_*) and 8.24% (using *H_exp_*). That is, in contrast to the other two genomes the genes within the genome of *K. racemifer* are distributed less even than expected by chance.

**Figure 6:**
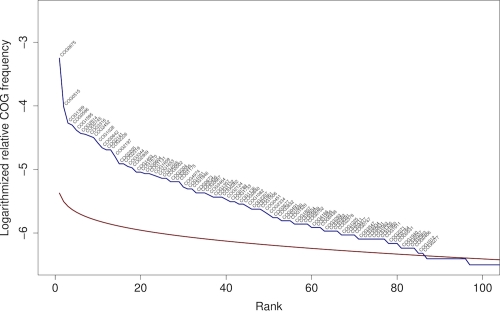
Relative frequencies of the 100 most frequent COGs in the genome of *K. racemifer* (blue line) compared to their expected frequency as estimated using the broken-stick distribution (red line). Over-represented COGs are labeled.

**Figure 7:**
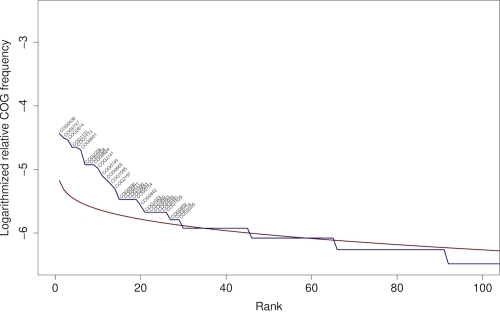
Relative frequencies of the 100 most frequent COGs in the genome of *S. thermophilus* (blue line) compared to their expected frequency as estimated using the broken-stick distribution (red line). Over-represented COGs are labeled.

**Figure 8:**
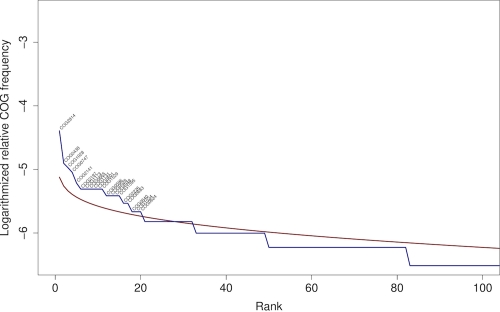
Relative frequencies of the 100 most frequent COGs in the genome of *T. roseum* (blue line) compared to their expected frequency as estimated using the broken-stick distribution (red line). Over-represented COGs are labeled.

Figure 6 compares the relative frequencies of the COGs in the genome of *K. racemifer* compared to their expected frequency. More than 80 COGs were judged as over-represented by this comparison, considerably more than in the genomes of *S. thermophilus* [33; Figure 7] and *T. roseum* ([15]; Figure 8). A closer look onto the 20 most over-represented COGs in *K. racemifer*, *S. thermophilus* and *T. roseum* revealed differences between the three organisms. Not surprisingly the genes coding transposases (COG0675; by far the most frequent one), integrases (COG3316) and resolvases (COG2452) can be found among the over-represented COGs in *K. racemifer* (Figure 6).

Our analyses also showed that genes belonging to the category COG3344 are over-represented in the genome of *K. racemifer*. COG3344 represents retron type reverse transcriptases, which are found in group II introns. Group II introns are large catalytic RNA molecules that act as mobile genetic elements [47]. They were first identified in mitochondria and chloroplast genomes, but with the increasing number of bacterial genome sequencing projects, the number of group II intron sequences in the databases also increased. Dai and Zimmerly reported in 2003 that a quarter of the sequenced bacterial genomes contain group II introns [48,49]. By using the IMG-ER platform [36] we calculated that approximately one third of the 2,727 sequenced bacterial genomes contain group II introns. In the genome of *K. racemifer*, 34 genes coding reverse transcriptases could be identified, all of them having the same domain structure with the reverse transcriptase domain followed by a maturase-specific domain and the C-terminal HNH-endonuclease domain.

## References

[1] Cavaletti L Monciardini P Bamonte R Schumann P Rohde M Sosio M Donadio S New lineage of filamentous, spore-forming, Gram-positive bacteria from Soil. Appl Environ Microbiol 2006; 72:4360-4369 10.1128/AEM.00132-06 16751552 PMC1489649

[2] Validation list No. 114. Int J Syst Evol Microbiol 2007; 57:433-434 10.1099/ijs.0.65052-0 17329763

[3] Yabe S Aiba Y Sakai Y Hazaka M Yokota A. *Thermosporothrix hazakensis* gen. nov., sp. nov., isolated from compost, description of *Thermosporotrichaceae* fam. nov. within the class *Ktedonobacter* Cavaletti et al. 2007 and emended description of the class *Ktedonobacteria*. Int J Syst Evol Microbiol 2010; 60:1794-1801 10.1099/ijs.0.018069-0 19767365

[4] Altschul SF Gish W Miller W Myers EW Lipman DJ Basic local alignment search tool. J Mol Biol 1990; 215:403-410 2231712 10.1016/S0022-2836(05)80360-2

[5] DeSantis TZ Hugenholtz P Larsen N Rojas M Brodie EL Keller K Huber T Dalevi D Hu P Andersen GL Greengenes, a Chimera-Checked 16S rRNA Gene Database and Workbench Compatible with ARB. Appl Environ Microbiol 2006; 72:5069-5072 10.1128/AEM.03006-05 16820507 PMC1489311

[6] Porter MF. An algorithm for suffix stripping. *Program: electronic library and information systems* 1980; **14**:130-137.

[7] Lee C Grasso C Sharlow MF Multiple sequence alignment using partial order graphs. Bioinformatics 2002; 18:452-464 10.1093/bioinformatics/18.3.452 11934745

[8] Castresana J Selection of conserved blocks from multiple alignments for their use in phylogenetic analysis. Mol Biol Evol 2000; 17:540-552 10742046 10.1093/oxfordjournals.molbev.a026334

[9] Stamatakis A Hoover P Rougemont J A rapid bootstrap algorithm for the RAxML Web servers. Syst Biol 2008; 57:758-771 10.1080/10635150802429642 18853362

[10] Hess PN De Moraes Russo CA An empirical test of the midpoint rooting method. Biol J Linn Soc Lond 2007; 92:669-674 10.1111/j.1095-8312.2007.00864.x PMC7110036 32287391

[11] Pattengale ND Alipour M Bininda-Emonds ORP Moret BME Stamatakis A How many bootstrap replicates are necessary? Lect Notes Comput Sci 2009; 5541:184-200 10.1007/978-3-642-02008-7_13

[12] Swofford DL. PAUP*: Phylogenetic Analysis Using Parsimony (*and Other Methods), Version 4.0 b10. Sinauer Associates, Sunderland, 2002.

[13] Liolios K Chen IM Mavromatis K Tavernarakis N Hugenholtz P Markowitz VM Kyrpides NC The Genomes OnLine Database (GOLD) in 2009: status of genomic and metagenomic projects and their associated metadata. Nucleic Acids Res 2010; 38:D346-D354 10.1093/nar/gkp848 19914934 PMC2808860

[14] Kiss H Nett M Domin N Martin K Maresca JA Copeland A Lapidus A Lucas S Berry KW Glavina Del Rio T Complete genome sequence of the filamentous predatory bacterium *Herpetosiphon aurantiacus* type strain (114-95^T^). Stand Genomic Sci 2011; (In press). 10.4056/sigs.2194987 PMC3368417 22675585

[15] Wu D Raymond J Wu M Chatterji S Ren Q Graham JE Bryant DA Robb F Colman A Tallon LJ Complete genome sequence of the aerobic CO-oxidizing thermophile *Thermomicrobium roseum.* PLoS ONE 2009; 4:e4207 10.1371/journal.pone.0004207 19148287 PMC2615216

[16] Pati A LaButti K Pukall R Nolan M Glavina Del Rio T Tice H Cheng JF Lucas S Chen F Copeland A Complete genome sequence of *Sphaerobacter thermophilus* type strain (S 6033^T^). Stand Genomic Sci 2010; 2:49-56 10.4056/sigs.601105 21304677 PMC3035262

[17] Kiss H Cleland D Lapidus A Lucas S Glavina Del Rio T Nolan M Tice H Han C Goodwin L Pitluck S Complete genome sequence of '*Thermobaculum terrenum'* type strain (YNP1^T^). Stand Genomic Sci 2010; 3:153-162 10.4056/sigs.1153107 21304745 PMC3035366

[18] Field D Garrity G Gray T Morrison N Selengut J Sterk P Tatusova T Thomson N Allen MJ Angiuoli SV The minimum information about a genome sequence (MIGS) specification. Nat Biotechnol 2008; 26:541-547 10.1038/nbt1360 18464787 PMC2409278

[19] Garrity G NamesforLife. BrowserTool takes expertise out of the database and puts it right in the browser. Microbiol Today 2010; 37:9

[20] Woese CR Kandler O Wheelis ML Towards a natural system of organisms: proposal for the domains *Archaea, Bacteria*, and *Eucarya.* Proc Natl Acad Sci USA 1990; 87:4576-4579 10.1073/pnas.87.12.4576 2112744 PMC54159

[21] Garrity GM, Holt JG. Phylum BVI. *Chloroflexi* phy. nov. *In*: Boone DR, Castenholz RW, Garrity GM (*eds*), Bergey's Manual of Systematic Bacteriology, second edition, vol. 1 (The *Archaea* and the deeply branching and phototrophic *Bacteria*) Springer, New York, 2001, p. 427-446.

[22] Hugenholtz P Stackebrandt E Reclassification of *Sphaerobacter thermophilus* from the subclass *Sphaerobacteridae* in the phylum *Actinobacteria* to the class *Thermomicrobia* (emended description) in the phylum *Chloroflexi* (emended description). Int J Syst Evol Microbiol 2004; 54:2049-2051 10.1099/ijs.0.03028-0 15545432

[23] BAuA Classification of bacteria and archaea in risk groups. http://www.baua.de. TRBA 2010; 466:112

[24] Ashburner M Ball CA Blake JA Botstein D Butler H Cherry JM Davis AP Dolinski K Dwight SS Eppig JT Gene Ontology: tool for the unification of biology. Nat Genet 2000; 25:25-29 10.1038/75556 10802651 PMC3037419

[25] Klenk HP Göker M En route to a genome-based classification of *Archaea* and *Bacteria*? Syst Appl Microbiol 2010; 33:175-182 10.1016/j.syapm.2010.03.003 20409658

[26] Wu D Hugenholtz P Mavromatis K Pukall R Dalin E Ivanova NN Kunin V Goodwin L Wu M Tindall BJ A phylogeny-driven genomic encyclopaedia of *Bacteria* and *Archaea*. Nature 2009; 462:1056-1060 10.1038/nature08656 20033048 PMC3073058

[27] List of growth media used at DSMZ: http//www.dsmz.de/microorganisms/media_list.php

[28] Gemeinholzer B Dröge G Zetzsche H Haszprunar G Klenk HP Güntsch A Berendsohn WG Wägele JW The DNA Bank Network: the start from a German initiative. Biopreservation and Biobanking 2011; 9:51-55 10.1089/bio.2010.0029 24850206

[29] The DOE Joint Genome Institute www.jgi.doe.gov

[30] Han C, Chain P. Finishing repeat regions automatically with Dupfinisher. in Proceeding of the 2006 international conference on bioinformatics & computational biology. Edited by Hamid R. Arabnia & Homayoun Valafar, CSREA Press. June 26-29, 2006: 141-146.

[31] Sims D Brettin T Detter JC Han C Lapidus A Copeland A Glavina Del Rio T Nolan M Chen F Lucas S Complete genome sequence of *Kytococcus sedentarius* type strain (strain 541^T^). Stand Genomic Sci 2009; 1:12-20 10.4056/sigs.761 21304632 PMC3035214

[32] Phrap and Phred for Windows. MacOS, Linux, and Unix. http://www.phrap.com

[33] Lapidus A, LaButti K, Foster B, Lowry S, Trong S, Goltsman E. POLISHER: An effective tool for using ultra short reads in microbial genome assembly and finishing. AGBT, Marco Island, FL, 2008.

[34] Hyatt D Chen GL LoCascio PF Land ML Larimer FW Hauser LJ Prodigal: prokaryotic gene recognition and translation initiation site identification. BMC Bioinformatics 2010; 11:119 10.1186/1471-2105-11-119 20211023 PMC2848648

[35] Pati A Ivanova NN Mikhailova N Ovchinnikova G Hooper SD Lykidis A Kyrpides NC GenePRIMP: a gene prediction improvement pipeline for prokaryotic genomes. Nat Methods 2010; 7:455-457 10.1038/nmeth.1457 20436475

[36] Markowitz VM Ivanova NN Chen IMA Chu K Kyrpides NC IMG ER: a system for microbial genome annotation expert review and curation. Bioinformatics 2009; 25:2271-2278 10.1093/bioinformatics/btp393 19561336

[37] NCBI Complete Microbial Genomes http://www.ncbi.nlm.nih.gov/genomes/lproks.cgi

[38] Schneiker S Perlova O Kaiser O Gerth K Alici A Altmeyer MO Bartels D Bekel T Beyer S Bode E Complete genome sequence of the myxobacterium Sorangium cellulosum. Nat Biotechnol 2007; 25:1281-1289 10.1038/nbt1354 17965706

[39] Wang XJ Yan YJ Zhang B An J Wang JJ Tian J Jiang L Chen YH Huang SX Yin M Genome sequence of the Milbemycin-producing bacterium *Streptomyces bingchenggensi.* J Bacteriol 2010; 192:4526-4527 10.1128/JB.00596-10 20581206 PMC2937363

[40] Copeland A Lapidus A Glavina Del Rio T Nolan M Lucas S Chen F Tice H Cheng JF Bruce D Goodwin L Complete genome sequence of *Catenulispora acidiphila* type strain (ID 139908^T^). Stand Genomic Sci 2009; 1:119-125 10.4056/sigs.17259 21304647 PMC3035231

[41] Nolan M Sikorski J Jando M Lucas S Lapidus A Glavina Del Rio T Chen F Tice H Pitluck S Cheng JF Complete genome sequence of *Streptosporangium roseum* type strain (NI 9100^T^). Stand Genomic Sci 2010; 2:29-37 10.4056/sigs.631049 21304675 PMC3035251

[42] Demharter W Hensel R Smida J Stackebrandt E *Sphaerobacter thermophilus* gen. nov., sp. nov. A deeply rooting member of the actinomycetes subdivision isolated from thermophilically treated sewage sludge. Syst Appl Microbiol 1989; 11:261-266

[43] Skerman VBD McGowan V Sneath PHA, eds. Approved Lists of Bacterial Names. Int J Syst Bacteriol 1980; 30:225-420 10.1099/00207713-30-1-225

[44] Auch AF von Jan M Klenk HP Göker M Digital DNA-DNA hybridization for microbial species delineation by means of genome-to-genome sequence comparison. Stand Genomic Sci 2010; 2:117-134 10.4056/sigs.531120 21304684 PMC3035253

[45] Auch AF Klenk HP Göker M Standard operating procedure for calculating genome-to-genome distances based on high-scoring segment pairs. Stand Genomic Sci 2010; 2:142-148 10.4056/sigs.541628 21304686 PMC3035261

[46] Legendre P, Legendre L. Numerical Ecology. 2nd edn. Elsevier, Amsterdam, 1998.

[47] Martínez-Abarca F Toro N Group II introns in the bacterial world. Mol Microbiol 2000; 38:917-926 10.1046/j.1365-2958.2000.02197.x 11123668

[48] Dai L Zimmerly S ORF-less and reverse-transcriptase-encoding group II introns in archaebacteria, with a pattern of homing into related group II intron ORFs. RNA 2003; 9:14-19 10.1261/rna.2126203 12554871 PMC1370365

[r49] 49. Kuever J, Rainey FA, Widdel F. Family I. *Desulfurellaceae* fam. nov. In: Brenner DJ, Krieg NR, Staley JT Garrity GM (eds), Bergey's Manual of Systematic Bacteriology, second edition, vol. 2 (The *Proteobacteria*), part C (The Alpha-, Beta-, Delta-, and *Epsilonproteobacteria*), Springer, New York, 2005, p. 923.

